# P-1796. A dual-mechanism model incorporating efflux pumps and resistance genes to predict multi-drug resistance in Klebsiella pneumoniae

**DOI:** 10.1093/ofid/ofaf695.1965

**Published:** 2026-01-11

**Authors:** Gabor Fidler, Mara Couto-Rodriguez, Heather L Wells, Sol Rey, Tiara Rivera, John C Papciak, Ford Combs, Caitlin Otto, Lorenzo Uccellini, Christopher E Mason, Niamh B O’Hara, Dorottya Nagy-Szakal, David C Danko

**Affiliations:** Biotia, Eger, Heves, Hungary; Biotia Inc., Brooklyn, New York; Biotia, Eger, Heves, Hungary; Biotia Inc., Brooklyn, New York; Biotia, Eger, Heves, Hungary; Biotia Inc., Brooklyn, New York; Biotia Inc, Washington DC, District of Columbia; Biotia Inc., Brooklyn, New York; Biotia inc, Long Island City, NY; Biotia Inc., Brooklyn, New York; Biotia Inc., Brooklyn, New York; Biotia Inc., Brooklyn, New York; Biotia, Eger, Heves, Hungary

## Abstract

**Background:**

Antimicrobial resistance (AMR) is a global threat projected to become a leading cause of death by 2050. Persistent selective pressure from antimicrobial overuse has driven the adaptation of resistant strains. While many AMR pathways are mediated by the presence of AMR genes (ARGs), efflux pumps (EPs) represent an often overlooked contributor to multi-drug resistance (MDR). *Enterobacterales* such as *Klebsiella pneumoniae* contain chromosomal EPs and are identified as a critical threat on the WHO Bacterial Priority Pathogens List because of growing rates of MDR. Our goal is to leverage EP regulatory sequences with known ARG markers to better predict AMR from urinary tract infection (UTI) clinical isolates using next-generation sequencing.

**Methods:**

We collected approximately 3000 genomic sequences with known phenotypes from the Bacterial and Viral Bioinformatics Resource Center (BV-BRC), identifying common ARGs with AMRFinder-plus. EPs and global regulators were characterized by aligning to a custom set of EP operons and global regulatory elements derived from type strains. Variant enrichment analysis was conducted to identify mutations in EP regulatory sequences that are significantly associated with resistant phenotypes. A machine learning model was developed and trained using the ARGs and enriched EP variants, then cross-validated using 369 clinical UTI isolates from our in-house strain collection.

**Results:**

We examined aminoglycoside resistance in *K. pneumoniae*, typically mediated by ARGs such as aminoglycoside-modifying enzymes and 16S rRNA methyltransferases. A model including only ARGs predicted gentamicin resistance with 73% accuracy. Adding major efflux pumps (AcrAB-TolC, AcrD, OqxAB) and global regulators (SoxS, MarA, BaeSR) improved accuracy to 89%, suggesting a synergistic role of ARGs and efflux systems in resistance.

**Conclusion:**

Incorporating EPs and regulatory sequences into AMR predictive models substantially increased sensitivity beyond ARGs alone. We identified signatures which might contribute to elevated EP capacity, and might be important genomic factors in resistance. We are continuing to adapt similar methods for additional resistance types found in *K. pneumoniae*, including fluoroquinolones and extended spectrum beta lactamases (ESBLs).

**Disclosures:**

Gabor Fidler, PhD, Biotia Inc: Employee Mara Couto-Rodriguez, MS, Biotia: Employee Heather L. Wells, MPH, PhD Candidate, Biotia: Employee Sol Rey, BS, Biotia: Employee Tiara Rivera, B.S., Biotia: Employee John C. Papciak, BS, Biotia: Employee Ford Combs, PhD, Biotia: Employee Caitlin Otto, PhD, D(ABMM), Biotia: Employee Lorenzo Uccellini, PhD, Biotia: Employee Christopher E. Mason, PhD, Biotia: Board Member Niamh B. O'Hara, PhD, Biotia: Employee Dorottya Nagy-Szakal, MD PhD, Biotia: Employee David C. Danko, Ph.D., Biotia: Employee

